# Rumor surveillance methods in outbreaks: A systematic literature review

**DOI:** 10.34172/hpp.2021.03

**Published:** 2021-02-07

**Authors:** Simin Salehinejad, Parya Jangipour Afshar, Vahidreza Borhaninejad

**Affiliations:** ^1^Medical Informatics Research Center, Institute for Futures Studies in Health, Kerman University of Medical Sciences, Kerman, Iran; ^2^Department of Biostatistics and Epidemiology, Faculty of Public Health, Kerman University of Medical Sciences, Kerman, Iran; ^3^Social Determinants of Health Research Center, Institute for Futures Studies in Health, Kerman University of Medical Sciences, Kerman, Iran

**Keywords:** Rumor, Misinformation, Public health surveillance, Disease outbreak

## Abstract

**Background:** The spreading of health-related rumors can profoundly put society at risk, and the investigation of strategies and methods can efficiently prevent the dissemination of hazardous rumor is necessary, especially during a public health emergency including disease outbreaks. In this article we review the studies that implicated the surveillance system in identifying rumors and discuss the different aspects of current methods in this field.

**Methods:** We searched PubMed, EMBASE, Scopus, and Web of Science databases for relevant publications in English from 2000 to 2020. The PICOS approach was used to select articles, and two reviewers extracted the data. Findings were categorized as a source of rumors, type of systems, data collection, and data transmission methods. The quality of the articles was assessed using the Mixed Method Appraisal Tool (MMAT) checklist.

**Results:** Five studies that presented the methods used for rumor detection in different outbreaks were included in the critical appraisal process. Findings were grouped into four categories: source of rumors, type of systems, data collection, and data transmission methods. The source of rumors in most studies was media, including new social and traditional media. The most used data collection methods were human-computer interaction technique, and automatic and manual methods each were discussed in one study. Also, the data transmission method was asynchronous in the majority of studies.

**Conclusion:** Based on our findings, the most common rumor detection systems used in the outbreaks were manual and/or human-computer methods which are considered to be time-consuming processes. Due to the ever-increasing amount of modern social media platforms and the fast-spreading of misinformation in the times of outbreaks, developing the automatically and real-time tools for rumor detection is a vital need.

## Introduction

In recent years, despite many advances in health and vaccine availability, emerging and re-emerging epidemics threatens community health. Severe acute respiratory syndrome (SARS), influenza A (H1N1), avian flu, Ebola virus, Middle East respiratory,^1^ and recent COVID-19 are among the best examples of such epidemics.

The public and media reactions during an outbreak period are different. When emergencies occur, people seek information more than usual. Invalid and unclear information not controlled by the government and the media may lead to psychological and emotional tensions, public panic, and economic loss within in a community.^2,3^ But, if there are clear and useful information and a valid channel for information dissemination among the government, the media, and the public, the level of social panic may be curbed and the expansion and spread of adverse effects in the event may be prevented. At the same time, a unique information interactive platform can better establish the credibility of the government and the media.^4^

It has been observed that the spread of misinformation and rumors regarding natural disasters and crises has increased.^5^ As an instance, Towers et al found that the news media may help spread fear and misinformation during Ebola epidemics in 2014.^6^ Also, during the SARS pandemics, there were network media information chaos, rumors, and public panic. There was also a high volume of misinformation about overbuying of medical-related products and the soaring prices of these products, which confused the community and caused many problems during disease control and prevention periods.^7^ Also, harmful rumors negatively affected people’s health during the outbreak of the infectious diseases such as SARS in 2003.^8^ Previous studies have highly emphasized the role of rumors’ management in critical periods of public health, like the Chernobyl nuclear accident in 1986, and the Ebola outbreak in Uganda in 2011.^9,10^ Although the management and monitoring of such rumors seems to be vital, identifying the quick and accurate sources of rumors, due to its complicated process and dynamic changes is highly challenging.^11^ The information diffusion in social sites opens many research trends like detection of misinformation and/or rumor checking, recognition of social bots, monitoring the spread of fake news, and prediction of future diffusion and source detection of rumors, as well.^12^ The results of a recent study on the use of health information in Senegal showed the necessity of investigating research methods for addressing health rumors.^13^ In an effort to develop such methods, Ma et al proposed two recursive neural models based on bottom-up and top-down tree-structured neural networks for rumor representation learning and classification.^14^

Besides, many efforts have been made to detect social media rumors by analyzing their content and social context using machine learning techniques.^15^ A new study, conducted by Liu and Xu, surveys user features in an online media platform for the detection of rumors. They developed a new information propagation model based on a different user representation and modeling method. Applying the new method, we can differentiate rumors from credible messages by observing distinctions in their respective propagation patterns in social media.^16^ All of these studies, therefore, shed light to a firm conclusion on the role of surveillance as the most efficient and effective way of handling rumors in outbreaks. Although the existing studies on rumor detection and surveillance focus on the theoretical modeling of classification and identification of rumors, there is still a need to understand the realistic methods used in previous outbreaks and epidemics.

There is a scarcity in the research done on the detection of rumors in outbreaks. To our knowledge, this is the first study that attempted to identify methods used for rumor detection in previous epidemics. Therefore, this study aimed to identify the methods and/or tools used for rumor detection and surveillance during outbreaks and to discuss their advantages and disadvantages.

## Material and Methods

This study was a systematic review of studies performed on the development of rumor surveillance systems during outbreaks and epidemics in the world.

### Information sources

We searched PubMed, EMBASE, Web of sciences, and Scopus databases for relevant papers in English. We searched the databases in November 2020 and included the related articles published from 2000 to 2020.

### Strategy search

n searching these databases, we used a mixture of medical subject headings(MeSH) and keywords. Three groups of key terms were used: (A) key terms denoting rumor spreading, including rumor, gossip, misinformation, (B) intervention related terms (e.g. outbreaks OR epidemics), and (C) outcome related terms (surveillance OR detection OR gossip OR misinformation).

We used two different strategies to extract relevant articles from the databases, and the results of the two strategies were then combined. To search the databases, advanced search functions were used as follows: first, we used “OR” to combine terms in each group A, B and C separately; then, we combined results from the two groups using the “AND” operator to accumulate all the studies about the rumor in outbreaks. We also searched for grey literature using the first 200 hits from Google Scholar and examined the references of the included studies to identify other potentially relevant studies. We used identical search terms and combined them in different ways (See Appendix 1).

### Eligibility criteria

Only the studies conducted on the problem of rumor spreading in outbreaks were considered for the review. Special emphasis was given to the interventional studies that developed a rumor surveillance system and applied it in an actual outbreak. Non-original and non-English papers, and the articles that focused on proposing and/or modeling a system, and those that evaluated such systems in drill or simulation were excluded from our study.

### Quality assessment

The quality of studies was evaluated using the Mixed Methods Appraisal Tool (MMAT)^17^ via answering two questions for all types of study, 5 questions for quantitative descriptive studies, and 5 questions for qualitative studies by two of the authors (See Appendix 2).

### Data selection, extraction, and analysis

After searching databases, two reviewers independently screened the articles first based on title and abstract. The full texts of all potentially eligible articles were retrieved and independently assessed for eligibility by two authors. Then, according to the predeﬁned inclusion and exclusion criteria, relevant articles were selected. Disagreements between the reviewers were resolved by a third reviewer. Finally, two reviewers extracted relevant information for each article. They abstracted the characteristics of eligible studies in a Microsoft Excel form. The characteristics were area, type of study, type of outbreaks, sources of rumor, type of surveillance system, data transfer method, and data collection method. Extracted data were summarized narratively and presented in a structured table, which were then analyzed using descriptive statistics.

## Results

### Study selection

**Figure 1:**
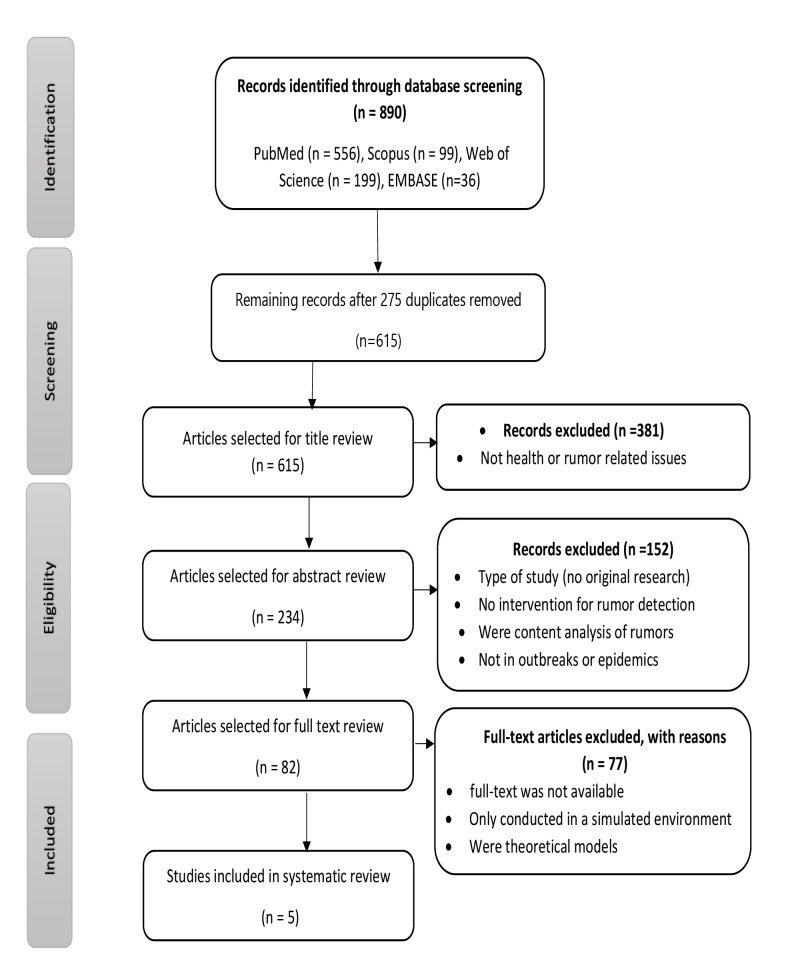
PRISMA flow chart for systematic literature review.

A total of 890 articles were initially retrieved according to the patients/problem, intervention, comparison, and outcome (PICO) search strategy. After removing the duplicates, 615 articles remained for screening. Most of the articles did not meet the inclusion criteria and only five articles were included in the final analysis (Figure 1).

### Study characteristics

Publication dates of the included articles ranged from 2005 to 2018. The literature on this topic began to show its presence in 2005 with an original article aimed to develop and implement a rumor surveillance system for avian influenza. All articles (n=5) were research-based and employed various methodologies. In terms of quality, all five articles were at a good level and met all MMAT criteria (one qualitative and four quantitative descriptive studies).

The studies included in this review had to match the predetermined criteria according to the patients/problem, intervention, comparison, outcome, and study design (PICOS) approach. Criteria for inclusion and exclusion are specified in Table 1.

### Geographical focus

Most of the literature has focused on African nations (n=3). One study discussed the use of social media in the countries affected by Zika, without a specific geographical focus. The other one, with a specific geographic focus, addressed the rumor surveillance system in the western pacific region (WPR), including 37 countries around the world.

### Types of outbreaks

The use of social media for informing about infectious diseases in outbreak situations was another common thread within this theme. The most specific type of outbreaks that addressed independently in the reviewed studies was influenza (two out of the five articles). One article specifically addressed more than one outbreak. One of these articles focused on the use of a rumor surveillance system within the recent Zika outbreak in 2016. Another article highlighted the development of the Surveillance and Outbreak Response Management System (SORMAS) based on the findings from the Nigeria’s 2014 Ebola outbreak. (Table 2)

### Sources of rumor surveillance

A focus on the sources of rumor surveillance was the most prominent theme identiﬁed in the literature. One article addressed social media as a source of rumors which specifically focused on the Twitter platform. Two articles mentioned other forms of media including the traditional media such as newspapers, TV, radio, and websites. Detecting rumors from community, health workers, and health facilities was highlighted in two of five studies. (Table 2)

### Type of surveillance system

The focus of most rumor surveillance systems was on human-computer interaction methods (n=3). Two papers specifically addressed the event-based surveillance (EBS) system, which was reported to require rapid detection, reporting, confirmation, and assessment of rare and new health events that can affect public health. These systems were integrated into the routine surveillance system. In another study, a software tool named SORMAS was used to support health workers in efﬁcient handling of the infectious diseases’ outbreak situations, such as Ebola.

Another study designated a rumor surveillance officer to develop and implement the rumor surveillance system for avian influenza. This officer actively assessed media sources and email-based public health discussions and regularly contacted the World Health Organization (WHO) network to identify rumors. It was categorized as a manual method. The automatic method was only used in one of the studies. Machine learning technology for tracking health misinformation was used in the 2016 Zika outbreak.

### Data transfer method

Our results showed that the data transmission method in three studies was asynchronous, within which data transfer took place over a while. On the contrary, synchronous (Real-time) methods were discussed in two studies. In this method, the time interval of data transmission is constant.

## Discussion

Exposure to misinformation and rumors may harm those who receive and believe them. In this study, the major source of rumors was found to be social and traditional media, followed by the rumors come from community and health workers.

As seen in the recent outbreaks of measles, Zika virus, and Ebola, the public is exposed to a large amount of information from both official channels, such as WHO and local authorities, and unofficial channels, such as newspapers and social media.^23,24^ Banakar et al revealed that social media, like WhatsApp, Telegram, and Instagram as well as the national media such as TV and radio were the primary sources of the Covid-19 news for the participants, and print media was less common sources.^25^ A reason for such a difference might be that the acquisition of information from social media platforms is more time-saving and less-costly than from the conventional news media such as newspapers or television. Besides, chatting and sharing information is easy on social media.^26^

### Methods for rumor surveillance

Different methods have been proposed for rumor detection and surveillance in previous outbreaks. According to our results in this review, in two studies, EBS was established along with the existing indicator-based surveillance systems for rumor surveillance. Design and implementation of such systems to retrieve and process the daily stream of misinformation is required for monitoring unofficial sources from the web regarding a wide range of health-related threats. Several EBS systems are currently monitoring unofficial sources on the web regarding a wide range of health threats.^27^ The EBS system is a simple-to-use and low-cost strategy that forms a cornerstone of public health surveillance and response, particularly in low-resource countries. Such systems use a combination of humans and computers for rumor and other event surveillance. Rumors and associated information are collected from different sources by health and/or surveillance officers are entered into Microsoft Excel 2013 for timely reporting and response.

As an instance of a rumor surveillance system, developed during the avian influenza outbreak in 2004, an officer actively assessed media sources and email-based public health discussions and contacted the WHO network, in a regular basis, to identify rumors. In this method, each rumor was followed up via an email or a telephone request to the relevant WHO country office to investigate its veracity.

An advantage of such a system is its low establishing cost, but it seems that the reporting time is high, especially in the situations of severe outbreaks when real-time response is critical. In the reviewed studies, the average time for rumor reporting was at a range from 3.8 to 10 days. Timely detection of health rumors can help public health ofﬁcials in tackling the issue before extensive spreading. Delay in reacting to a rumor, may increase its damage through rapid spreading of its harmful misconceptions. For example, during the Ebola outbreak, a rumor that circulated on the Internet presented “drinking salty water” as an effective protective measure, which led to several deaths in the population.^28^ This may also reflect the surveillance of rumors at different levels of the health system. While in two studies^19,22^ rumors surveillance was at the national and international levels, in another study^18^ the surveillance was conducted at the health centers level, which is much closer to the community. So, the reporting time is much shorter than those at the national level.

Machine learning is another method for health-related rumor discovery that was used for social media surveillance. Ghenai et al. building a machine learning tool for tracking the health misinformation on Twitter during Zika outbreak. .^20^ Compared to traditional media, social media is harder to be monitored, tracked, and analyzed. Public health institutions such as the WHO introduced social media as a crucial part of monitoring while the surveillance of a health crisis.^29^ Due to the nature of the textual data and their fast spreading in social media, designing tools that automatically identify rumors and assess their accuracy increased in recent years.^30^ Machine learning methods emerged as key players in rumor detection on online social media.

Qazvinian et al in 2011 proposed a supervised machine learning method for judging the relevance of new tweets to the known set of rumors. Their results showed that particularly known rumors can be retrieved with high accuracy after training a machine learning classifier for each rumor. Due to the ever-increasing amount of multimedia information on social media, many efforts have been made to automatically defeat online rumors. Such a process is conducted through mining the rich content provided on the open network with machine learning techniques, like neural network methods.^31^ Recently, deep neural networks are proposed to automatically learn and fuse multi-modal features for rumor detection. Compared to the works that leverage the traditional methods, such networks can significantly improve the performance of the systems.^32^

The third method is developing an electronic tool such as a mobile and web-based application that improves data collection, situation assessment, and coordination of response measures in outbreaks. SORMAS is a mobile-based and open-source system that uses a cloud service provider, and is accessible via both personal computers and smartphones. This system is applicable for outbreak management and better routine surveillance of all infectious diseases. A section of this application is a rumor table which contains information about all rumors that may be concerned to one or more cases, while detecting rumors reported by someone, an event, or a place. In some countries, the national public health authorities are preparing to deploy SORMAS in response to the COVID-19 outbreak.^33^

In contrast to SORMAS, there are other mobile-based and web-based software that are exclusively used for monitoring the people who had made contact with Ebola cases and do not address other aspects of outbreak response, like case ﬁnding and rumor surveillance.^21^ For example, many software tools are especially developed to monitor the emerging infectious diseases^34,35^and their associated rumor detection in social media and online sources. TweetCred,^36^ Hoaxy,^37^ and SUPER^38^ are examples of these type of tools.

SORMAS can improve the processes and help health workers in faster reactions compared to the manual or traditional methods. The advantages of this application are their acceptability,^39^ low development costs and lead-in times, and the support that it provides for real-time rumor management, contact-tracing, case management, and surveillance.^40^ In the study of Yavlinsky et al, several mobile-based outbreak management systems were analyzed, but only SORMAS was satisfying and fulfilled all the assessment criteria.^41^

To sum up, the rumors spreading on social media can severely inﬂuence people’s daily life. However, to react to the ever-increasing rumors, a majority of the rumor management methods still depend on manual efforts, including human experts or users. In this circumstance, the research on automatic rumor detection is fascinating. Most of the rumors in social media are multimodal which result in difﬁculties when they are managed using traditional detection methods. Also, data transmission in the traditional methods is asynchronous and on the basis of store-and-forward systems which allow for multiple component interactions to occur. However, the effect of the time gap may need information refreshing during the process. Also, the synchronic model and the real-time methods are increasingly used in the new methods such as machine learning systems to support immediate interaction or at least response within a short time.^42^ Cost-saving is another advantage of such methods.^43^

This paper gives an overview of rumor surveillance methods. Among all the existing methods, some use human-computer methods for rumor surveillance. Compared to the manual methods, these computerized methods prepare better results with higher efficiency, as they reduce labor costs and their associated findings are more accurate. These characteristics of the computerized methods ensure the real-time availability of the most data for everyone involved in the outbreak containment, and allow public health researchers and health practitioners to respond to the rumors with a targeted and rapid reaction. To our knowledge, this was the first study that systematically identified the methods used for rumor detection used in the outbreaks, and assessed their effectiveness.

Due to the nature of the study, our study had several limitations. The number of articles that applied a system for rumor surveillance during outbreaks and included in our study was low. Despite the increasing research on different methods of rumor detection, there were few studies that used this method in outbreaks. So, we had limitations in comparing the effect of different methods on a real condition. In general, the findings of this study indicated that much more efforts should be made to identify the effectiveness of different rumor surveillance methods during outbreaks.

## Conclusions

In this study, we reviewed the methods used for rumor detection in the outbreaks. Our findings provided lessons for health care organizations, health care professionals, researchers, and the general public on how to optimize the rumor detection and surveillance methods in recent COVID-19 outbreak. Due to the ever-increasing use of social media, and considering the broad and rapid deployment of information in wide and fast spread infectious disease like COVID-19, developing the new methods such as mobile applications, neural networks, and machine learning systems that can automatically detect and find the sources of misinformation in a timely manner is pivotal.

## Acknowledgements

We are grateful to institute for futures studies in health in Kerman University of Medical Sciences, for their support in conducting this research.

## Funding

This work was supported by Vice Chancellor of Research and Technology, Kerman University of Medical Sciences, Kerman, Iran.

## Competing interests

The authors have no conflicts of interest to declare.

## Ethics approval

This study received ethical approval from the vice chancellor for research of the Kerman University of Medical Sciences (IR.KMU.REC.1399.005).

## Authors’ contributions

SS contributed in searching, reviewing the literature, summarizing the results, and writing up the manuscript. PJA also contributed to reviewing the literature and summarizing the results. SS and VB contributed to the design and finalize the manuscript.

**Table 1:** PICOS criteria for inclusion and exclusion of studies

**Criteria**	**Inclusion**	**Exclusion**
Problem	studies discussed the rumor spreading in outbreaks	Studies not discussed rumor spreading in health-related situation
Intervention	Rumor detection or surveillance methods/ systems	Studies not discussed any rumor surveillance method
Comparison	No comparator	
Outcomes	How effective are the different methods (e.g. time and cost-saving)	Studies without defined outcomes
Study design	Original quantitative and qualitative research studies were included if they examined the use or establishment of rumor surveillance or detection methods in previous epidemics or outbreaks	Review articles, case reports, commentaries, and editorials.

**Table 2:** Description and Analysis of Included Articles

Authors	Date of publication	Affected area	Type of study	Type of outbreaks	Type of system	Source of rumor	Data transfer method	Data collection method
Toyama et al^18^	2015	North-western Ethiopia	Quantitative description	Measles, Suspected rabies, anthrax, etc	EBS system	Community members HPs workers and between HC and other health facilities and schools	Asynchronous	Human-computer interaction
Samaan et al^19^	2005	Western pacific regional	Quantitative description	Avian influenza H5N1	Rumor surveillance system	Media sources included journalists visiting WPRO and Web sites for television networks and newspapers	Asynchronous	Completely human (manual)
Ghenai et al^20^	2017	All country affected by zika	Quantitative description	Zika fever	Machine learning	Twitter	Synchronous (Real-time)	Computer (automatic)
Perscheid et al^21^	2018	Nigeria	Qualitative	Ebola	SORMAS software tool	Community and health facilities e.g., via phone calls, or from Informants	Synchronous (Real-time)	Human-computer interaction
Dagina et al^22^	2013	Papua New Guinea	Quantitative description	Influenza	EBS system	Health workers, nongovernmental organizations, embassies, media, and the general public	asynchronous	Human-computer interaction

Abbreviation: EBS, event-based surveillance; SORMAS, Surveillance and Outbreak Response Management System; HP, health posts; HC,health centers

**Appendix 1:** The search terms used in the PICOS search

	**Pubmed**	**Web of sciences**	**Scopus**	**EMBASE**
Problem	"rumor"[Title/Abstract]) OR "rumour"[Title/Abstract] OR "misinformation"[Title/Abstract] OR "gossip"[Title/Abstract]	(rumor) *OR* TOPIC: (rumour) *OR* TOPIC: (gossip) *OR* TOPIC: (misinformation)	( TITLE-ABS-KEY ( *rumor*) OR TITLE-ABS-KEY ( *rumour*) OR ( TITLE-ABS-KEY ( *gossip*) OR TITLE-ABS-KEY ( *misinformation*)	rumor:ti,ab,kw OR rumour:ti,ab,kw OR misinformation:ti,ab,kw OR gossip:ti,ab,kw
Intervention	("disease outbreaks"[MeSH Terms] OR "epidemics"[MeSH Terms])	TOPIC: (outbreaks) OR TOPIC: (epidemics)	( TITLE-ABS-KEY ( *outbreaks* ) OR TITLE-ABS-KEY ( *epidemics* )	outbreak:ti,ab,kw OR epidemic:ti,ab,kw OR misinformation:ti,ab,kw OR gossip:ti,ab,kw
Comparison	n/a	n/a	n/a	n/a
Outcomes	(("Public Health Surveillance "[ MeSH Terms] OR "detection"[Title/Abstract] OR "mange"[Title/Abstract] OR "understand"[Title/Abstract])	(surveillance) OR TOPIC: (detection) *OR* TOPIC: (mange) *OR* TOPIC: (understand)	( TITLE-ABS-KEY ( *surveillance*) OR TITLE-ABS-KEY ( *detection*) OR ( TITLE-ABS-KEY ( *mange*) OR TITLE-ABS-KEY ( *understand*)	surveillance:ti,ab,kw OR detect:ti,ab,kw OR manage:ti,ab,kw OR understand:ti,ab,kw

*PICO (patients/problem, intervention, comparison, outcome)

**Appendix 2:** Quality assessment of included study

**Category of study designs**	**Methodological quality criteria**	**Toyama**	**Samaan**	**Ghenai**	**Perscheid**	**Dagina**
Screening questions (for all types)	S1. Are there clear research questions?	yes	yes	yes	yes	yes
	S2. Do the collected data allow to address the research questions?	yes	yes	yes	yes	yes
Qualitative	1.1. Is the qualitative approach appropriate to answer the research question?				yes	
	1.2. Are the qualitative data collection methods adequate to address the research question?				yes	
	1.3. Are the findings adequately derived from the data?				yes	
	1.4. Is the interpretation of results sufficiently substantiated by data?				yes	
	1.5. Is there coherence between qualitative data sources, collection, analysis and interpretation				yes	
Quantitative descriptive	4.1. Is the sampling strategy relevant to address the research question?	yes	yes	yes		yes
	4.2. Is the sample representative of the target population?	yes	yes	yes		yes
	4.3. Are the measurements appropriate?	yes	yes	yes		yes
	4.4. Is the risk of nonresponse bias low?	yes	yes	yes		yes
	4.5. Is the statistical analysis appropriate to answer the research question	yes	yes	yes		yes
